# Effect of diabetes-specific nutrition formulas on satiety and hunger hormones in patients with type 2 diabetes

**DOI:** 10.1038/s41387-019-0093-x

**Published:** 2019-09-24

**Authors:** Adham Mottalib, Martin J. Abrahamson, David M. Pober, Rani Polak, Ahmed H. Eldib, Shaheen Tomah, Sahar Ashrafzadeh, Osama Hamdy

**Affiliations:** 1 Joslin Diabetes Center, Harvard Medical School, Boston, MA 02215 USA; 2 Department of Medicine, Lahey Hospital and Medical Center, Burlington, MA 01805 USA; 3 Beth Israel Deaconess Medical Center, Harvard Medical School, Boston, MA 02215 USA; 4 Spaulding Rehabilitation Hospital, Harvard Medical School, Boston, MA 02129 USA

**Keywords:** Type 2 diabetes, Type 2 diabetes, Nutrition, Obesity

## Abstract

**Objectives:**

Diabetes-specific nutritional formulas (DSNFs) are frequently used by patients with type 2 diabetes (T2D) as part of nutrition therapy to improve glycemic control and reduce body weight. However, their effects on hunger and satiety hormones when compared to an isocaloric standardized breakfast are not fully understood. This study aims to evaluate the postprandial effects of two DSNFs—Glucerna (GL) and Ultra Glucose Control (UGC)—versus oatmeal on selected satiety and hunger hormones.

**Method:**

After an overnight fast, 22 patients with T2D (mean age 62.3 ± 6.8 years, A1C 6.8 ± 0.7%, body weight 97.4 ± 21.3 kg, and BMI 33.2 ± 5.9 kg/m²) were given 200 kcal of each meal on three separate days. Blood samples for amylin, cholecystokinin (CCK), ghrelin, glucagon, leptin, and peptide-YY (PYY) were collected at baseline and 30, 60, 90, 120, 180, and 240 min after the start of each meal. Incremental area under the curve (iAUC_0-240_) for each hormone was calculated.

**Results:**

iAUC_0-240_ for glucagon and PYY were significantly higher after GL and UGC than after oatmeal (*p* < 0.001 for both). No difference was observed between the three meals on postprandial amylin, CCK, ghrelin, and leptin hormones.

**Conclusions:**

Intake of DSNFs significantly increases secretion of PYY and glucagon, two important satiety hormones. While subjective satiety was not directly evaluated, the increased effect on satiety hormones may partially explain the mechanism of body weight loss associated with DSNF use.

## Introduction

Nutrition therapy and increased physical activity are first-line therapies for patients with type 2 diabetes (T2D)^1^. Diabetes-specific nutritional formulas (DSNFs) may be used as a component of medical nutrition therapy (MNT) to help in improving glycemic control and reducing body weight^2,3^. There is evidence that the integration of meal replacement formulas into an MNT plan leads to better compliance with nutrition therapy and greater weight loss compared to patients on an isocaloric MNT plan^4^. Recently, the American Diabetes Association included DSNFs in its clinical practice recommendations for lifestyle management^5^. However, the mechanisms by which DSNFs lead to weight loss and improved blood glucose control are not fully understood.

Regulation of appetite is a complex process that involves intricate pathways of hormonal and neuronal signaling^6,7^. We previously reported that in comparison to an isocaloric oatmeal breakfast, two commercially available DSNFs significantly increased production of postprandial glucagon-like peptide-1 (GLP-1) hormone^8^. GLP-1 is an incretin hormone which increases insulin secretion and suppresses glucagon secretion, leading to enhanced glycemic control and increased satiety as a result of DSNF consumption^9,10^.

This study was conducted to explore the effects of two commercially available DSNFs: Glucerna (GL, Abbott Nutrition Inc., Columbus, OH, USA) and Ultra Glucose Control (UGC, Metagenics, Inc., Aliso Viejo, CA, USA) versus a common breakfast food, namely oatmeal (oatmeal, Quaker Old Fashioned Oats, Quaker Oats Co., Chicago, IL, USA) on several other satiety and hunger hormones in overweight and obese patients with T2D.

## Subjects and methods

### Study design and subjects

This cross-over, three-way, and open-label, ancillary study was conducted in accordance with the Helsinki Declaration and was approved by the institutional Committee on Human Studies. All participants signed a written informed consent. The study was registered at ClinicalTrials.gov (Identifier: NCT02691481). Eligible subjects were patients with T2D for ≥3 months, ages 18 to 75 years, body mass index (BMI) > 25 kg/m^2^, and glycated hemoglobin A1C (HbA_1c_) ≥ 6.5%. Patients using diabetes or cholesterol-lowering medications had to be on stable doses of these medications for ≥3 months. Exclusion criteria included pregnancy or lactation, use of insulin or GLP-1 analogs, history of bariatric surgery, gastroparesis, and malabsorption syndrome. Twenty-five subjects were enrolled in the study, of which 22 subjects completed all study visits. One subject dropped out prior to the first study visit for personal reasons. Two other subjects dropped out after the first visit due to inconvenience of frequent blood sampling. Data from those subjects were excluded from statistical analysis. Mean age of the remaining 22 subjects (±SD) was 62.3 ± 6.8 years, diabetes duration was 9.5 ± 9.8 years, and HbA_1c_ was 6.8 ± 0.7%. Baseline characteristics of the study subjects are summarized in Table 1.

**Table 1:** Characteristics of study subjects

Variable	
Sex	
Male	12 (54.6%)
Female	10 (45.5%)
Age (years)	62.3 ± 6.8
Weight (kg)	97.4 ± 21.3
BMI (kg/m^2^)	33.2 ± 5.9
Diabetes duration (years)	9.5 ± 9.8
A1C (%)	6.8 ± 0.7

*N* = 22. Sex *n* (%), remaining variables are mean ± standard deviation

### Analyzed hormones

Amylin is a satiety hormone that is co-secreted with insulin from pancreatic β-cells^11^. Its secretion induces satiety by stimulating the brainstem to slow gastric emptying and decrease gastric secretions^12,13^. Cholecystokinin (CCK) is a satiety hormone secreted by enteroendocrine cells in the duodenum and jejunum^14^. Its actions include the promotion of gallbladder contraction, inhibition of gastric acid secretion, and slowing of gastric emptying^15^. Glucagon is secreted by pancreatic alpha cells and induces satiety through the vagus nerve^13^. Leptin is secreted by adipose tissue and stimulates satiety centers in the hypothalamus^16^. Peptide-YY_3-36_ (PYY) is secreted by enteroendocrine L-cells^6^ and acts as a satiety signal to the hypothalamus while reducing gastric acid secretion and gastrointestinal motility^17^. Ghrelin is a hunger hormone secreted mainly by the stomach^18^. Its stimulates gastrointestinal motility and gastric acid secretion^19^.

### Study procedures

Subjects were asked to come for three visits with a washout period between visits of at least two days. All visits were completed over three weeks. Subjects were instructed to come for each visit after fasting overnight for at least 8 h and were asked to withhold their diabetes and cholesterol-lowering medications on the morning of the visit. In random visit order, each subject was asked to ingest one of the three tested meals (GL, UGC and oatmeal) for breakfast. All meals were 200 kcal each. GL was provided in a 237 mL (8 fl oz) bottle; UGC was prepared by dissolving 200 kcal powder in 296 mL (10 fl oz) of water; and oatmeal was prepared by adding water to 56 g of dry oats and cooking the mixture on a stove for 5–10 min. No milk, sugar, or sweetener was added. Macronutrient composition of the three breakfast meals is shown in Table 2.

**Table 2:** Nutrition information of the three breakfast meals

	Oatmeal		Glucerna		Ultra Glucose Control	
	Amount	% DV	Amount	% DV	Amount	% DV
Serving size	53.3 (g)	NA	237 (mL)	NA	56 (g)	NA
Energy (kcal)	200	10	200	10	200	10
Total fat (g)	4	6.7	7	11	7	11
% Energy	18	–	32	–	32	–
Saturated fat (g)	0	0	0.5	3	1	5
Monounsaturated fat (g)	1.3	–	5.2	–	4.5	–
Total carbohydrates (g)	36	12	26	9	27	9
% Energy	72	–	52	–	54	–
Dietary fiber (g)	5.3	20	3	12	3	12
Protein (g)	6.7	13.4	10	20	15	30
% Energy	13	–	20	–	30	–

%DV: percentage daily values were calculated based on a 2000 kcal diet. Oatmeal (Quaker Oats Co., Chicago, IL, USA); Glucerna (Abbott Nutrition Inc., Columbus, OH, USA); Ultra Glucose Control (Metagenics Inc., Aliso Viejo, CA, USA)

For safety, blood glucose was measured at the beginning of each visit. If blood glucose was between 70–300 mg/dL, a venous line was inserted and a baseline blood sample was drawn. This was followed by consumption of the test meal within 3–5 min. Blood samples were collected at 30, 60, 90, 120, 180, and 240 min from the start of each meal. Blood samples were tested for serum active amylin, CCK, ghrelin, glucagon, leptin, and PYY. After collection of the last sample, subjects were given a snack and were instructed to take their regular medications.

### Statistical analyses

Values for all measured variables are presented as mean ± SD or standard error of the mean (SEM). Study data were analyzed using SAS statistical software (SAS Institute Inc., Cary, NC, USA). Analysis was performed using linear mixed effects models to model the covariance structure arising from the repeated measures design. Where overall *F*-tests were significant, pairwise differences of the treatment means were tested with *t*-tests using Tukey’s p-value adjustments. Outcomes were defined as area under the curve between 0 and 240 min for measured variables over time (AUC_0–240_) calculated using the trapezoidal formula^20^. Incremental AUC between 0 and 240 min (iAUC_0–240_) was calculated using the same formula but representing the area above the fasting level.

## Results

**Fig. 1:**
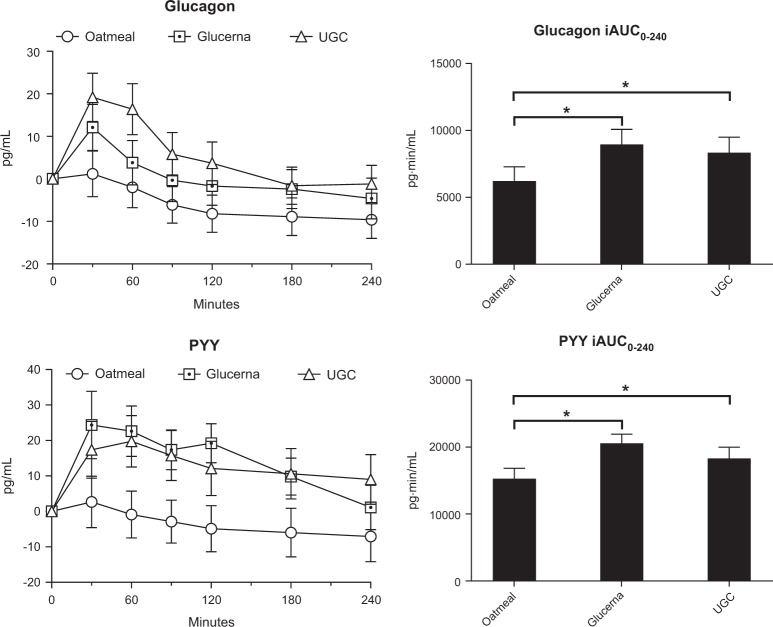
Adjusted serum concentrations of glucagon and peptide YY (PYY) in 22 subjects with type 2 diabetes after intake of 200kcal of oatmeal, Glucerna, and Ultra Glucose Control (UGC). Values are mean ± SEM. *p < 0.0001

Mean fasting serum glucagon for oatmeal, GL, and UGC were similar (35.8 ± 4.4, 41.9 ± 4.7, and 34.5 ± 4.6 pg/mL respectively). Glucagon iAUC_0-240_ was significantly higher after GL and UGC compared to oatmeal (*p* < 0.0001 for both); however, there was no difference in glucagon iAUC_0–240_ between GL and UGC (Fig. 1).

Mean fasting serum PYY for oatmeal, GL, and UGC were similar (72.2 ± 7.2, 76.5 ± 7.8, and 68.7 ± 8.3 pg/mL respectively). PYY iAUC_0–240_ was significantly higher after GL and UGC compared to oatmeal (*p* < 0.0001 for both); however, there was no difference in PYY iAUC_0–240_ between GL and UGC (Fig. 1).

**Fig. 2:**
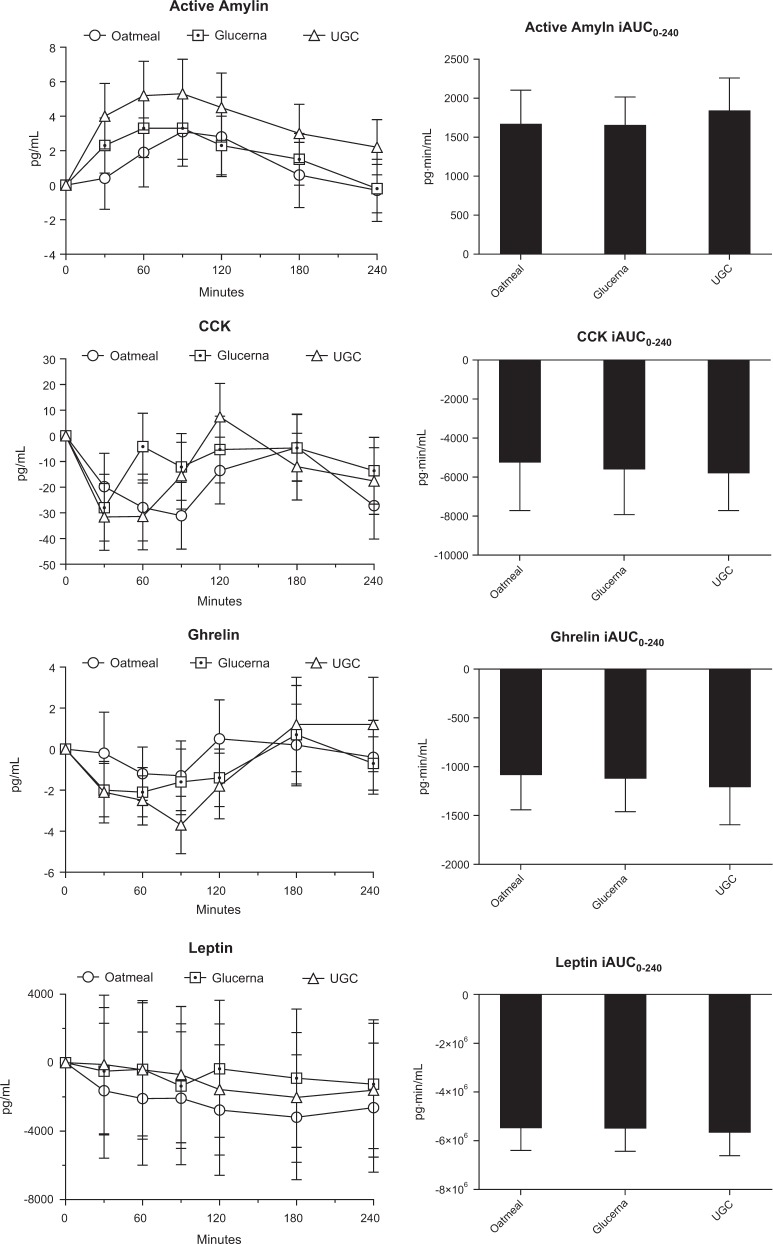
Adjusted serum concentrations of active amylin, cholecystokinin, ghrelin, and leptin in 22 subjects with type 2 diabetes after intake of 200kcal of oatmeal, Glucerna, and Ultra Glucose Control (UGC). Values are mean ± SEM. CCK cholecystokinin. Incremental area under the curve was not different between meals for all four variables

Mean fasting serum active amylin for oatmeal, GL, and UGC were similar (10.5 ± 1.6, 9.9 ± 1.4, and 8.9 ± 1.3 pg/mL respectively). Active amylin iAUC_0-240_ showed no significant differences between meals (*p* = 0.076) (Fig. 2).

Mean fasting serum CCK for oatmeal, GL, and UGC were similar (38.8 ± 18.7, 33.8 ± 18.2, and 37.8 ± 16.0 pg/mL respectively). CCK iAUC_0–240_ showed no significant differences between meals (*p* = 0.85) (Fig. 2).

Mean fasting serum ghrelin for oatmeal, GL, and UGC were similar (9.1 ± 1.6, 10.0 ± 1.6, and 10.3 ± 2.1 pg/mL respectively). Ghrelin iAUC_0–240_ showed no significant differences between meals (*p* = 0.82) (Fig. 2).

Mean fasting serum leptin for oatmeal, GL, and UGC were similar (25224.9 ± 4273.6, 23649.5 ± 3820.1, and 24790.2 ± 4012.4 pg/mL respectively). Leptin iAUC_0–240_ showed no significant differences between meals (*p* = 0.87) (Fig. 2).

## Discussion

In the Look AHEAD (Action for Health in Diabetes) study^3^ and other shorter studies^4,21^, use of DSNFs as part of a hypocaloric nutrition therapy was associated with weight reduction that was clearly associated with their frequency of use to replace calories or smaller meals. This study provides a mechanistic explanation of that effect, where two of the commercially available DSNFs showed significant increase in two essential weight-modulating hormones that contribute to satiety and increased energy expenditure. Both tested DSNFs increased PYY in comparison to isocaloric oatmeal. This study also showed that both DSNFs significantly stimulate glucagon secretion in comparison to isocaloric oatmeal. Glucagon affects glycemia and satiety. Despite its stimulatory effect on hepatic glucose production, glucagon hormone increases glucose metabolism, and energy expenditure^22,23^. In addition, glucagon indirectly stimulates satiety through an afferent signal from the hepatic branch of the vagus nerve^24^. These observations complement our previous observation that both DSNFs stimulate GLP-1 hormone production, another strong satiety hormone, in comparison to isocaloric oatmeal^8^.

Despite previous claims that all calories are created equal in their effect on body weight^25^, this study shows that different macronutrients have different effects on key satiety and weight-modulating hormones since all tested meals were of equal caloric content. The two studied DSNFs are higher in their protein and fat content and lower in their carbohydrate content than oatmeal (Table 2). It has been debated which macronutrient(s) elicit the highest postprandial PYY response. An earlier study favored fat in producing the highest PYY response^26^. However, more recent studies showed that protein induces the highest PYY response^27^ and carbohydrates induce the smallest effect^28^. Our results are also in line with previous studies that showed meals higher in both protein and fat content induce higher glucagon response compared to a carbohydrate-rich meal^24,29^.

Although both tested DSNFs stimulate two opposing weight-modulating hormones, GLP-1^8^ and glucagon, our findings suggest that the stimulatory effect of protein and fat within DSNFs is stronger on glucagon secretion than the inhibitory effect of GLP-1 on glucagon production. Postprandial amylin levels were marginally higher following ingestion of UGC compared to GL and oatmeal, but this difference was not statistically significant (*p* = 0.076). Furthermore, there were no differences in the postprandial effects of DSNFs on CCK, ghrelin, and leptin hormones. While these changes in satiety hormones provide an attractive potential explanation for the reported success of DSNFs in supporting weight loss, it is also possible that these changes in the satiety hormones, while statistically significant, may not be of sufficient magnitude to explain an effect on satiety that is large enough to interpret their role in improved weight loss.

The present study had several limitations which include the difference in texture between oatmeal and DSNFs. A previous study reported difference in satiety between solid and liquid meal replacements^30^. This study was powered to detect differences in glucose AUC_0–240_ rather than differences in the analyzed hunger and satiety hormones. Background diets of the study subjects were not controlled and their effect on the study outcomes is unknown. We aimed to minimize that effect by asking subjects to fast for at least 8 h before each visit. In addition, subjects completed all study visits within a three-week window.

In conclusion, this study shows that DSNFs significantly increase secretion of two satiety hormones; PYY and glucagon. This effect may be related to their specific macronutrient composition. While the effect of the three different meals on subjective satiety was not directly evaluated, results from this study may partially explain the mechanism of body weight reduction associated with DSNFs use.

## References

[1] Nathan DM (2009). Medical management of hyperglycaemia in type 2 diabetes mellitus: a consensus algorithm for the initiation and adjustment of therapy: a consensus statement from the American Diabetes Association and the European Association for the Study of Diabetes. Diabetologia.

[2] Elia M (2005). Enteral Nutritional Support and Use of Diabetes-Specific Formulas for Patients With Diabetes A systematic review and meta-analysis. Diabetes Care.

[3] Wadden TA (2011). Four-year weight losses in the Look AHEAD study: factors associated with long-term success. Obesity.

[4] Heymsfield S, Van Mierlo C, Van der Knaap H, Heo M, Frier H (2003). Weight management using a meal replacement strategy: meta and pooling analysis from six studies. Int. J. Obes..

[5] Association AD (2019). 5. Lifestyle Management: Standards of Medical Care in Diabetes—2019. Diabetes Car.

[6] Cummings DE, Overduin J (2007). Gastrointestinal regulation of food intake. J. Clin. Investig..

[7] Ahima RS, Antwi DA (2008). Brain regulation of appetite and satiety. Endocrinol. Metab. Clin. North Am..

[8] Mottalib A (2016). Impact of diabetes-specific nutritional formulas versus oatmeal on postprandial glucose, insulin, GLP-1 and postprandial lipidemia. Nutrients.

[9] Flint A, Raben A, Astrup A, Holst JJ (1998). Glucagon-like peptide 1 promotes satiety and suppresses energy intake in humans. J. Clin. Investig..

[10] Nadkarni P, Chepurny OG, Holz GG (2014). Regulation of glucose homeostasis by GLP-1. Prog. Mol. Biol. Transl. Sci..

[11] Lukinius A, Wilander E, Westermark GT, Engstrom U, Westermark P (1989). Co-localization of islet amyloid polypeptide and insulin in the B cell secretory granules of the human pancreatic islets. Diabetologia.

[12] Young AA (2012). Brainstem sensing of meal-related signals in energy homeostasis. Neuropharmacology.

[13] Woods SC, Lutz TA, Geary N, Langhans W (2006). Pancreatic signals controlling food intake; insulin, glucagon and amylin. Philos. Trans. R. Soc. Lond. Ser. B, Biol. Sci..

[14] Kissileff HR, Pi-Sunyer FX, Thornton J, Smith GP (1981). C-terminal octapeptide of cholecystokinin decreases food intake in man. Am. J. Clin. Nutr..

[15] Chaudhri O, Small C, Bloom S (2006). Gastrointestinal hormones regulating appetite. Philos. Trans. R. Soc. Lond. Ser. B, Biol. Sci..

[16] Myers MG, Cowley MA, Munzberg H (2008). Mechanisms of leptin action and leptin resistance. Annu. Rev. Physiol..

[17] le Roux CW, Bloom SR (2005). Peptide YY, appetite and food intake. Proc. Nutr. Soc..

[18] Cummings DE (2001). A preprandial rise in plasma ghrelin levels suggests a role in meal initiation in humans. Diabetes.

[19] Cummings DE (2006). Ghrelin and the short- and long-term regulation of appetite and body weight. Physiol. Behav..

[20] Pruessner JC, Kirschbaum C, Meinlschmid G, Hellhammer DH (2003). Two formulas for computation of the area under the curve represent measures of total hormone concentration versus time-dependent change. Psychoneuroendocrinology.

[21] Cheskin LJ (2008). Efficacy of meal replacements versus a standard food-based diet for weight loss in type 2 diabetes: a controlled clinical trial. Diabetes Educ..

[22] Jiang G, Zhang BB (2003). Glucagon and regulation of glucose metabolism. Am. J. Physiol. Endocrinol. Metab..

[23] Salem V (2016). Glucagon increases energy expenditure independently of brown adipose tissue activation in humans. Diabetes, Obes. Metab..

[24] Geary N (1990). Pancreatic glucagon signals postprandial satiety. Neurosci. Biobehav. Rev..

[25] Sacks FM (2009). Comparison of weight-loss diets with different compositions of fat, protein, and carbohydrates. N. Engl. J. Med..

[26] Essah PA, Levy JR, Sistrun SN, Kelly SM, Nestler JE (2007). Effect of macronutrient composition on postprandial peptide YY levels. J. Clin. Endocrinol. Metab..

[27] van der Klaauw AA (2013). High protein intake stimulates postprandial GLP1 and PYY release. Obesity.

[28] Cooper JA (2014). Factors affecting circulating levels of peptide YY in humans: a comprehensive review. Nutr. Res. Rev..

[29] Raben A, Agerholm-Larsen L, Flint A, Holst JJ, Astrup A (2003). Meals with similar energy densities but rich in protein, fat, carbohydrate, or alcohol have different effects on energy expenditure and substrate metabolism but not on appetite and energy intake. Am. J. Clin. Nutr..

[30] Tieken SM (2007). Effects of solid versus liquid meal-replacement products of similar energy content on hunger, satiety, and appetite-regulating hormones in older adults. Horm. Metab. Res..

